# Proceedings of Seventh Annual Meeting of the Ohio State Dental Society, Held at Columbus, O., Dec. 4, 1873

**Published:** 1873-11

**Authors:** 


### Proceedings of Societies.

### PROCEEDINGS OF SEVENTH ANNUAL MEETING OF THE OHIO STATE DENTAL SOCIETY, HELD AT COLUMBUS, O., DEC. 4, 1873.

The meeting was called to order at 11 A. M., by the Vice-President, I. Williams, in the absence of the President, and opened with prayer by Rev. Mr. Laidlaw ; a large number of the members answering to their names on the calling of the roll. The minutes of the previous meeting were read and approved.

Dr. Blount suggested that the president appoint some one in his place on the Committee on Membership, and that those desiring membership make application immediately after adjournment.

The President appointed Dr. McKinley.

Dr. J. Taft moved that a committee of three, consisting of the Secretary, Treasurer and one other member, be appointed to correct the roll of membership. The motion prevailed.

The President appointed Dr. Herriot as the additional member. Dr. J. Taft moved that a committee of three be appointed to consider the propriety of holding a "Quiz" during the session and to make such suggestions as they deem best; which motion prevailed.

The President appointed as [illegible] that committee, Drs. H. A. Smith, Cin'ti; A. A. Blount, [illegible] ringfield; and G. W. Keely.

On motion it was agreed t[illegible] morning session be from 9:30 A. M. to 12:30 P. M., the afte[illegible] oon session from 2 P. M.
P. M., and the evening session from 7:30 P. M. to 10:30 P. M. After discussion in regard to the propriety of holding an evening session, the matter was left with the Executive Committ-
ee to determine.

On motion of J. Taft, the Society adjourned until 2 o'clock P. M.

### AFTERNOON SESSION.

The Society reassembled at 2 o'clock. The minutes of the foıenoon meeting were read. The report of the Committee on Membership being called for, Dr. Porter stated that they were not yet ready to report.

The next topic, "The Burring Engine," was then taken up for discussion, Dr. H. A. Smith read an essay on this topic, which, on motion of Dr. Taft, was received and referred to the Committee on Publication.

The discussion of this topic was opened by Dr. Herriot and further participated in by Drs. Rosson, Jennings, Buffets, Siddall, J. H. Warner, C. R. Taft, Williams, Gray and Clancey.

On motion of Dr. Kelsey, the Society passed to the discussion of the next topic; viz, "The best means of preserving the eye-sight of the dental practitioner." Essays were read upon this topic by Drs. Porter and I. Williams, which, on motion, were received and referred to the Committee on Publication. The discussion was continued by Drs. Buffett, R. G. Warner, Kelsey, Herriot, Jennings, Rosson and J. M. Porter.

Dr. Porter from the Committee on Membership reported as suitable persons to become members of the Society: L. B. Welch, Wilmington; C. G. Von Bonhorst, Lancaster; J. H. Warner, Columbus; C. B. Knowlton, Elyria; J. F. Siddall, Oberlin; J. B. Beauman, Columbus; D. W. Clancey, Cincinnati; W. E. Hawley, Easton; N. C. Sine, Delaware; and C. R. Sabine, Troy. On motion of Dr. Taft, the report was received and the Society proceeded to ballot for the candidates.

Dr. Rehwinkle moved that the Secretary be authorized to cast an affirmative vote for those proposed for membership, in order to save time and expedite business.

Dr. J. Taft moved to amend, to provide that if there was no objection to the persons named. The amendment was accepted.

Dr. J. Taft suggested that the applicants retire from the room while the vote was being taken, which they did. He also suggested that each one be balloted for separately, so that if it was desired to ask any questions in regard to any candidate, they might have full opportunity to do so. The suggestion was acted upon.

Dr. Porter, Chairman of the Committee on Membership, at the request of Dr. Taft, made some remarks in regard to the examination of candidates, stating they must have either a diploma from a Dental College or a certificate from the State Board of Examiners, before they can be received as members of this Society. Dr. Smith, from the committee appointed to consider the propriety of holding a "quiz," reported that they recommended the holding of a quiz of half an hour's duration at the commencement of the evening sessions, and that Dr. Herriot conduct the same on the first evening, Dr. Harroun on the second evening and Dr. Kelsey on the third evening. On motion, the report was accepted and adopted.

Dr. Rosson suggested that the Executive Committee arrange for the holding the next meeting before leaving Columbus.

Dr. Rehwinkel coincided with this suggestion and further suggested that the parts assigned to the different members be given them, that they might know what they would have to do.

The discussion on "The best means of preserving the eyesight of the dental practitioner" was resumed by Drs. Rosson, J. H. Warner, Porter, H. A. Smith, Siddall, Rehwinkle and Herriot.

At the conclusion of the discussion the Society adjourned until 7 P. M.

FIRST DAY, EVENING SESSION.

The Society reassembled at 7 P. M. Half an hour was spent in a quiz, conducted by Dr. Herriot. The questions proposed were generally in reference to the burring engine.

The discussion on the second topic was again resumed and further discussed by Drs. C. R. Taft, Harroun, Beauman, Gray, Herriot, J. F. Siddall and J. H. Siddall.

Dr. Rosson offered a resolution of which he had given notice one year ago, providing for the nomination of more than one candidate for each office of the Society.

The resolution was discussed by Drs. Rosson, Warner, Kelsey, Jennings, Harroun and Siddall. The Secretary read a provision in the constitution of the Society, showing that the adoption of the resolution required a two-thirds vote of all the members present. The question being on the adoption of the resolution a rising vote was held, twenty-one members voting in the affirmative and none in the negative.

On motion, the meeting was adjourned to meet at 9 o'clock A. M., for clinics.

2D DAY, AFTERNOON SESSION.

The meeting reassembled at 2 o'clock P. M.

On motion of Dr. C. R. Taft, W. T. Wallace, a delegate from the Pennsylvania State Dental Society, being present was invited to participate in the proceedings of the Society.

On motion, it was resolved that Dr. D. C. Hawxhurst, ex-President of the Michigan Central Dental Association and Dr. W. K. Jones, delegate from the West Va. Dental Association be elected honorary members of this Society, and invited to participate in the discussions and bear to their respective societies the greetings of this body.

Dr. Porter offered the following :

*Resolved*, That five hundred copies of the Constitution, By-laws and Code of Ethics of this Society be published and distributed among the members of the Society, and that the Secretary furnish a copy to all persons joining the Society. and that all persons violating the Code of Ethics shall be expelled, and his name published in the DENTAL REGISTER and his county paper.

Dr. R. G. Warner moved to amend by making the number of copies one thousand instead of five hundred. The resolution as amended was adopted.

Dr. Porter also offered the following :

*Resolved*, that there be no clinics in operative dentistry at the future meetings of this Society. Adopted.

Dr. Porter from the Committee on Membership reported, recommending the following persons for membership in the Society : W. E. Jaques, Burton ; A. H. Johnson, Massillon ; Joseph Craig, Canton ; M. L. Wright, Chardon ; S. Vail, New Philadelphia ; S. W. Peasley, Norwalk ; C. F Stroud, Sandusky ; H. H. Newton, Cleveland ; L. G. Dills, Greenfield; Samuel Wagner, Gallion ; F. O. Jacobs, Coshocton ; L. D. Converse, Urbana ; D. Jones, North Fairfield ; J. E. Phelps, Chagrin Falls ; J. A. Umstot, Cadiz ; J. N. Custar, Westerville ; A. E. Griffin, Bellefontaine ; and H. Clark, Circleville.

On motion, the report was accepted and the candidates balloted for.

On motion, the Secretary was instructed to cast an affirmative ballot for the candidates, as each one was proposed.

On motion, the names of Dr. L. G. Dills, of Greenfield, and L. D. Converse. of Urbana, when called, were passed over for further investigation.

Dr. C. R. Taft, Chairman of the Committee appointed to revise the roll of membership, made report, stating that the Committee proceeded under a resolution heretofore adopted, which provided that a member who had failed to attend the meetings or to pay his dues for two years should be dropped, and could not be reinstated except upon the payment of all back dues. Dr. Taft here read a list of the names of the active members of the Society, as revised, showing one hundred working members.

Dr. Rosson stated that the state law, regulating the practice of dentistry, would go into effect on the first day of January, and he desired to know how the members felt in regard to it, whether they intended to enforce the law. Dr. H. A. Smith followed in some remarks, showing the determination on the part of members of the profession to enforce the law, and instanced the case being prosecuted by Dr. C. H. James, in Cincinnati.

Dr. Harroun moved that the Society set aside three hundred dollars to be used, if necessary, in assisting in the prosecution begun by Dr. James.

Dr. Harroun withdrew his motion for the introduction of a resolution by Dr. Porter, as follows:

*Resolved*, That the chair appoint a committee of three, whose duty it shall be to select one or more dentists in each county, as far as practicable, to report the names of dentists who may be practicing in violation of the law regulating the practice of dentistry, after the first day of January, 1873, in the counties in which they reside, also in adjoining counties, and see that the law be enforced.

The resolution was discussed by Drs. H. A. Smith, Harroun, Herriot, J. F. Siddal, J. H. Siddal, Porter and J. Taft.

Dr. J. Taft suggested that the resolution be modified, so that it might seem somewhat smoother in its phraseology.

After further remarks by Drs. J. Taft, H. A. Smith and L. Buffett, Dr. Smith moved to amend the resolution by striking out the words "and see that the law be enforced." He proposed that instead of this the names and professional standing of all such persons be reported at the next meeting. The motion prevailed.

Dr. J. H. Warner moved a reconsideration of the vote. A division was called for, and twenty-two members, a majority of those present, voted in favor of a reconsideration.

After other motions and suggestions, the whole question, on motion of Dr. J. Taft, was referred to a committee consisting of Drs. Porter, Rehwinkle and Kelsey, to report at the earliest convenience.

Dr. Harroun then renewed his motion. which was adopted. Dr. H. R. Smith, Dr. Wheeler and Dr. Harroun were appointed a committee to have the management of the fund of three hundred dollars. Dr. L. Buffett suggested it would be well to allow Dr. Smith to draw that amount if needed.

Dr. Harroun thought the suggestion a good one, and moved that Dr. Smith be empowered to draw on the fund of three hundred dollars to be set aside to defend the case referred to in Cincinnati. Motion adopted.

Dr. Rehwinkle presented the report of the Committee on th Rubber Question, and made some remarks in connection therewith.

On motion of Dr. Herriot, the report was accepted and the committee continued.

Dr. Herriot moved that the Committee be granted discretionary power, in regard to the amount of money to be expended in this matter to the extent of five hundred dollars. The motion was carried.

Dr. Beauman moved that the Society extend its thanks and gratitude to Dr. S. S. White, for the action he has taken in regard to the Rubber Question, and that it signify its readiness to do its part financially in the matter. Motion carried.

Dr. H. A. Smith moved that a committee of three, consistin[illegible] of Drs. Herriot, Warner and Beauman, be appointed to ob[illegible]in conditional subscriptions for this purpose, and convey [illegible] Dr. White the resolution of Dr. Beauman. Carried.

[illegible]r. Porter, from the Committee appointed in reference to t[illegible] dues owed by members offered the following :

*Resolved*, That all members of this Society, who shall neglect to pay their annual dues for two consecutive years, shall not be permitted to resume their membership without first paying all back dues.

The resolution was adopted.

On motion of Dr. Rosson, the discussion of the las

"The best means of preserving the eyesight of the dental practitioner," was resumed and farther participated in by Drs. J. Taft, J. H. Warner and Rehwinkle, when on motion the Society adjourned until 7 o'clock P. M.

2D DAY, EVENING SESSION.

The Society re-assembled at 7 P. M., The first half hour being devoted to a quiz conducted by Dr. Harroun, subject.—Proper Diagnosis of Diseases of the Mouth.

Dr. Herriot, from the Committee to which was entrusted the preparation of Resolutions, expressive of the feelings of the Society towards Dr. S. S. White, for his labors in the interests of the profession against the Goodyear Dental Vulcanite Company, reported the following :

*Resolved*, That we recognize the integrity of purpose and efficiency of service of Dr. S. S. White against the Goodyear Dental Vulcanite Company, and we most heartily thank him, for the conclusive evidence he has given us of the fraud practiced upon the Supreme Court of the United States by that Company, detrimental to the interests of the dentists in the Gardner Case ; and that we also tender to Dr. White, material aid in that suit, the funds to be placed at the disposal of the Dental Vulcanite Committee, if in their judgment they may be needed. The report was accepted. Dr. Horton moved that the resolutions be adopted, and that a copy signed by the officers of the Society be sent to Dr. White : which was agreed to, and the resolution adopted.

Dr. Herriot made a further report from the Committee, recommending the assessment of ten dollars on each member of the Society, if in the opinion of the Committee on the Rubber Question it be necessary to be used to defray expenses in fighting that Company. The recommendation was discussed by Drs. Rosson, Beauman, Harroun, Sidall and Horton and was unanimously adopted.

Dr. Kelsey offered the following :

*Resolved*, That this Society recommend to the licensees of the Goodyear Dental Vulcanite Company that they transact no further business with that Company until the question now before the United States Court, in regard to the Cumming's patent is finally settled. A division was called for and the resolution was adopted.

Dr. J. Taft moved that the law regulating the practice of Dentistry in the State of Ohio, be published in connection with the Code of Ethics. Motion agreed to.

The Committee appointed to report on the action to be taken by the dentists in reference to the Dental Law, reported as follows:

*Resolved*, That the Chair appoint a Judiciary Committee, whose duty it shall be to act upon all violations of the Code of Ethics, and to confer with a Committee of one or more dentists in each county, in regard to violations of the law regulating the practice of dentistry, and advise with the dentist or dentists in the county, to determine whether an active or passive course be pursued in relation to such violations of the law, after having learned through the County Committee the extent and nature of such violation.

The report was accepted and adopted.

Dr. Hawxhurst, ex-President of the Michigan Central Dental Society addressed the meeting upon "The effects of fetid breath upon the constitution." Dr. Kelsey moved that a vote of thanks be returned to Dr. Hawxhurst for his address, and that a copy of the paper be requested for publication. The motion was agreed to. Dr. Taft moved that the paper be discussed at the next session, which was agreed to.

The Committee on Membership recommended the following named persons for membership in the Society: P. A. Palmer, Marietta; E. B. Yager, Orville; F. H. Houghton, Columbus; M. Chapin, Akron; W. L. Hosie, Newton Falls; A. F. Emminger, Columbus; D. G. French, Xenia; G. D. Bellows, Medina; C. H. Bowles, Granville.

On motion, the Society then adjourned until 9:30 A. M.

### THIRD DAY, MORNING SESSION.

The Society reassembled at 9:30 A. M. The minutes were read and approved.

The President appointed as the Judiciary Committee, Drs. Herriot, Harroun and Porter.

Dr. Rawson moved that Dr. L. D. Converse, of Urbana, be admitted as a member of this Society, which motion was agreed to.

The Auditing Committee was then appointed.

Dr. L. Buffett moved that the name of Dr. J. N. Custar be passed upon ; a ballot being had, he was declared duly elected.

The Committee on Membership reported the following additional names : W. T. Wallace, Kingsville ; H. L. Crider, Lancaster ; J. C. Johnson, Granville.

Dr. C. R. Taft stated that the minutes showed that some persons had been elected members ot this Society, who had neither signed the constitution, nor paid their initiation fee, and asked for instructions as to whether their names should be placed on the roll of membership.

Dr. Rosson moved that the names of all such persons be dropped from the roll as if no action had been taken in their cases, which motion was agreed to.

Dr. Herriot moved that Dr. H. A. Smith be re-elected a member of the State Board of Examiners, which motion was agreed to.

Dr. L. Buffett moved that the President appoint a committee of two, to appoint delegates to the American Dental Association, which was agreed to.

The President appointed as said committee, Drs. Buffett and Warner.

On motion of Dr. Rosson, the Society resolved itself into committee of the whole for the purpose of nominating officers for the ensuing year, Dr. Jennings in the chair. The officers to be elected being President, two Vice-Presidents, Recording Secretary, Corresponding Secretary and Treasurer. The following nominations were made : Dr. Jennings nominated Dr. L. Buffett, of Cleveland, for President ; Dr. J. H. Warner nominated Dr. B. F. Rosson, of Troy, for First Vice-President ; Dr. Harroun nominated Dr. W. M. Herriot for Second Vice-President ; Dr. Rosson nominated Dr. C. R. Taft for Recording Secretary.

Dr. J. H. Warner nominated Dr. D. R. Jennings, for President ; Dr. Herriot nominated Dr. Harroun for First Vice President; also Dr. Hall Second Vice President ; Dr. Rosson nominated Dr. I. Williams for Treasurer ; Dr. Porter was nominated for Recording Secretary ; Dr. Herriot nominated Dr. C. R. Butler for Corresponding Secretary.

Dr. Rosson moved that the committee rise and proceed to the election of officers. Carried.

The first ballot for President resulted in no choice. Dr. Jennings then withdrew his name. Upon the second ballot, Dr. Buffett was declared duly elected President. For First Vice-President, Dr. B. F. Rosson was elected; for Second Vice-President Dr. Herriot was elected ; for Recording Secretary, Dr. C. R. Taft was elected ; for Corresponding Secretary, Dr C. R. Butler was elected ; for Treasurer, Dr. I. Williams, being the only candidate nominated, the Secretary was instructed to cast an affirmative ballot for him for that office and he was declared elected. The President appointed Drs. Harroun and Beauman to conduct the President elect to the chair. Upon taking the chair he was greeted with applause and made a few appropriate remarks.

Dr. I. Williams returned thanks to the members for their kindness and courtesy to him, as acting President during the sessions of the Society.

On motion of Dr. Rosson, a vote of thanks was tendered Dr. I. Williams, ex-Vice-President, who had presided during the session, for the impartial and able manner in which he had presided over the deliberations of the body during the three days just past, which was unanimously agreed to.

The paper of Dr. Hawxhurst was then taken up and the discussion upon it opened by Dr. Rosson, and further participated in by Drs. Harroun, Herriot, Kelsey, Porter, J. Taft, H. A. Smith, Hawxhurst and Rehwinkle.

A paper by Dr. Watt on Vital Force, was read by Dr. J. Taft.

On motion, a vote of thanks was tendered Dr. Watt for his paper. The paper was discussed by Drs. J. Taft and Hawxhurst.

The President appointed the following standing committees for the ensuing year :

On motion of Dr. Taft, a paper of Dr. Butler on "Dental Law" was referred to the Publication Committee. A paper of Dr. Way, of Sandusky, was presented by Dr. J. Taft.

On motion of Dr. Herriot, this paper was referred to the Publication Committee for publication, if they deemed best.

The Committee on Membership reported the name of Dr. W. F. Harbaugh, of Piqua, who was elected a member of the Society.

The Treasurer made his report showing a balance on hand of $241.17.

Dr. Taft moved that a committee of three be appointed to ascertain the number and locality of dentists in the State of Ohio, which was agreed to.

The chair appointed as that committee Drs. Will Taft, M. DeCamp and C. Palmer.

Dr. Herriot moved that the Publication Committee be instructed to publish the Constitution, By-laws, Code of Ethics and dental law in such form as they may think best, and send to all the dentists in the State.

Dr. Porter moved that a committee of three be appointed to draft a suitable amendment to the statute exempting dentists from jury duty, the same as physicians and ministers, which was agreed to.

The President appointed J. Taft, H. A. Smith and M. De Camp as such committee.

On motion, the Society adjourned until 2 o'clock P. M.

THIRD DAY, AFTERNOON SESSION.

The Society reassembled at 2 P. M., Dr. L. Buffett in the chair. The President named as the Judiciary Committee, Drs. W. M. Herriot, J. M. Porter and C. H. Harroun.

The Committee to select delegates to the American Dental Association reported through Dr. R. G. Warrer the following names: W. P. Horton, A. A. Blount, M. DeCamp, C. H. Harroun, C. B. Knowlton, M. C. Sine, I. Williams, L. D. Converse, A. E. Griffin, W. P. Hall, W. R. Lilly, J. M. Porter, J. H. Warner, H. W. Howe, F. H. Rehwinkle, A. T. Price, A. A. Newton and W. F. Harbaugh.

Dr. Porter moved that in case the suit pending in Cincinnati be decided in favor of the profession the result be published in the DENTAL REGISTER. Carried.

Dr. Buffett, Treasurer for the previous year, made his report.

Dr. Buffett moved that the janitor be paid five dollars for taking care of the hall during the sessions of the Society, carried.

Dr. Horton presented the report of the Examining Board and its doings for the past year. On motion of Dr. Herriot, the report was received and adopted.

In a further report of the finances, the statement was made that the Board had received at the June meeting $ 425 00

| | | |
|---|---|---|
| At the present meeting, Dec., | | 1700 00 |
| | | 2125 00 |
| Expenses at the June meeting | 213 25 | |
| " " " December meeting | 602 50 | |
| | 815 75 | |
| By amount paid Treasurer | 1309 25 | |
| | 2125 00 | |

Dr. Herriot moved that the funds be placed in the hands of the "Society for Savings," Cleveland, O., to the credit of the Ohio State Dental Society, motion agreed to.

Dr. J. H. Warner, on behalf the Auditing Committee, presented the account of I. Pugh for $20.00. On motion the account was accepted and ordered paid. Dr. Horton was, on motion, appointed to act as Treasurer *pro tem* to pay this account. On motion, the Society then adjourned until the first Tuesday of December, 1873.

